# A 28-Year-Old Man with Stridor and Dyspnea

**DOI:** 10.3390/jcm14051532

**Published:** 2025-02-25

**Authors:** Francesco Rocco Bertuccio, Davide Valente, Nicola Baio, Stefano Tomaselli, Laura Saracino, Gaetano Sciandrone, Alessandra Milanesi, Paolo Delvino, Veronica Codullo, Angelo Guido Corsico, Giulia Maria Stella

**Affiliations:** 1 Unit of Respiratory Disease, Cardiothoracic and Vascular Department, IRCCS Policlinico San Matteo, Viale Golgi 19, 27100 Pavia, Italy; f.bertuccio@smatteo.pv.it (F.R.B.); nicola.baio01@universitadipavia.it (N.B.); s.tomaselli@smatteo.pv.it (S.T.); l.saracino@smatteo.pv.it (L.S.); g.sciandrone@smatteo.pv.it (G.S.); a.corsico@smatteo.pv.it (A.G.C.); 2 Department of Internal Medicine and Pharmacology, University of Pavia, 27100 Pavia, Italy; 3 Radiology Institute, Fondazione IRCCS Policlinico San Matteo, 27100 Pavia, Italy; davide.valente@icsmaugeri.it; 4 PhD Experimental Medicine, Università di Pavia, 27100 Pavia, Italy; alessandra.milanesi01@universitadipavia.it; 5 Department of Internal Medicine and Therapeutics, University of Pavia, 27100 Pavia, Italy; v.codullo@smatteo.pv.it; 6 Division of Rheumatology, Fondazione IRCCS Policlinico San Matteo, 27100 Pavia, Italy; 7 School of Medicine, University of Milano-Bicocca, 20126 Milan, Italy; paolodelvino@unimib.it; 8 Rheumatology Unit, IRCCS San Gerardo dei Tintori, 20900 Monza, Italy

**Keywords:** vasculitis, granulomatosis with polyangiitis, tracheobronchial stenosis, multidisciplinary approach, rigid bronchoscopy

## Abstract

**Background:** Tracheobronchial stenosis is a significant complication in granulomatosis with polyangiitis (GPA), a systemic vasculitis that primarily affects the upper respiratory tract, kidneys, and lungs. The involvement of the tracheobronchial tree in GPA leads to airway narrowing, which can result in severe respiratory symptoms and increased morbidity, often requiring prompt diagnosis and management to prevent life-threatening airway obstruction. **Method:** We present the case of a 28-year-old male with mild exertional dyspnea, stridor, and retropharyngeal sputum. Clinical investigations revealed subglottic and bronchial concentric stenosis with granulomatous inflammation. A diagnosis of granulomatosis with polyangiitis (GPA) with isolated tracheobronchial stenosis (TBS) was confirmed. **Results:** Given the severity of airway obstruction, multidisciplinary management was initiated, combining rigid bronchoscopy with systemic immunosuppressive therapy. Post-intervention follow-up demonstrated significant airway improvement and maintained remission after two years. **Conclusions:** This case highlights TBS as a potentially debilitating GPA manifestation requiring a combination of systemic and endoscopic therapies. Further studies are needed to optimize therapeutic approaches and improve outcomes in GPA-associated TBS.

## 1. Case Presentation

A young 28-year-old patient came to our attention for mild dyspnea on exertion, stridor, persistent nasal crusting and retropharyngeal constant sputum occurring for a few months. He denied smoking and had no exposure to potentially toxic inhaled agents. He was previously diagnosed with subclinical hypothyroidism in chronic autoimmune thyroiditis and atopic dermatitis. Due to worsening of symptoms, further diagnostic investigations were started.

At presentation, the patient appeared in no acute distress. Vital signs were normal. Oxygen saturation was 98% on room air. Respiratory examination revealed scattered bilateral coarse crackles that mobilized with coughing, compatible with airway secretions and stridor. Cardiac and abdominal examinations were unremarkable. Joint pain, skin lesions, and peripheral sensory–motor neurological defects were all absent. In addition, there were no signs of nasal or auricular chondritis.

Laboratory analysis revealed normal full blood count, creatinine (1.02 mg/dL), C-reactive protein, and liver function tests. Urinalysis was unremarkable, with a 24 h proteinuria of 80 mg and no signs of active glomerulopathy on microscopic examination of the urine sediment. The autoimmune panel, including ANA, ENA, dsDNA, and ANCA was negative, with normal complement levels. Negative antibodies were found for ribonucleoprotein, rheumatoid factor, cyclic citrullinated protein, scleroderma-100, anti-Sjögren-syndrome-related antigens A and B, anti-Jo, and anti-centromere. The lymphocyte subset panel was within normal range, as well as the immunoglobulin levels. He also had a negative HIV status, QuantiFERON test, and hepatitis panel.

**Figure 1:**
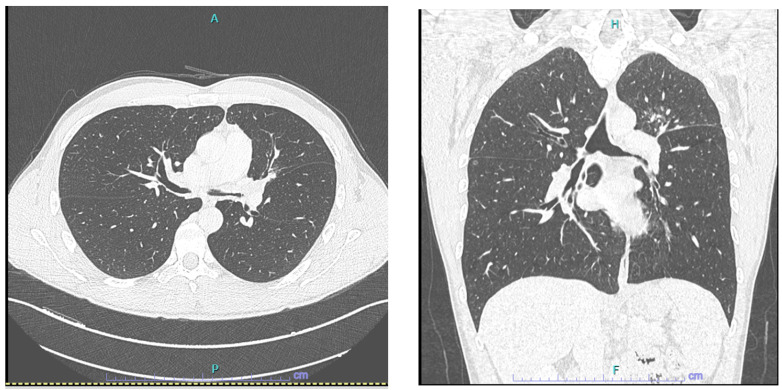

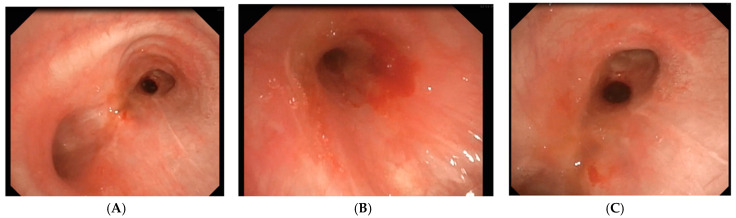
From left to right: left main bronchus obstruction (CT scan axial projection). Left main bronchus obstruction (CT scan coronal projection). (**A**–**C**) From left to right: enlarged tracheal carina lined with thickened mucosa but with regular surface; concentric stenosis of the left main bronchus; concentric caliber reduction in the right main bronchus.

Subsequently, a computed tomography (CT) scan of the neck and chest was performed; the latter reported modest, irregular thickening of the mucosal wall on the left bronchial segments. Irregular wall thickening and caliber reduction were found in the upper lobar bronchus, which was completely obstructed (Figure 1). No abnormalities were observed in the right bronchial hemisystem on the right, in the absence of significant lymphadenopathy. No bony erosions were observed at the sinus CT scan.

Given the radiological findings, the patient underwent a bronchoscopy. The latter revealed subglottic concentric inflammatory stenosis, combined with enlarged tracheal carina lined with thickened mucosa (Figure 1, panel A) and concentric caliber reduction in the main right bronchus (Figure 1, panel C). The right upper lobar bronchus was of normal size, while concentric stenosis of the bronchus intermedius made the middle and lower lobar segments unexplorable. On the left, conspicuous concentric stenosis of the main bronchus was identified (Figure 1, panel B). A periprocedural biopsy was performed at the level of the carina, with histologic examination showing extensive erosion/ulceration and histiocytic inflammation (CD68+) with granulomatous features. Multiple microbiological investigations, including bacterial, fungal, and mycobacterial stains and cultures, were negative.

## 2. Clinical Course

Considering clinical, radiological, and histological findings, the diagnosis of granulomatosis with polyangiitis with isolated tracheobronchial stenosis was confirmed.

Given the severity of the subglottic and bronchial stenosis, the case was submitted to multidisciplinary discussion with rheumatologists, pneumologists and otorhinolaryngologists, with indication to combine endoscopic and systemic treatment; consequently, rigid bronchoscopy was scheduled for endoscopic intervention.

Dilatation with progressive rigid instrument up to 13.2 mm was performed to treat the concentric inflammatory subchordal tracheal stenosis (which showed marked inflammation and easily bleeding mucosa with a residual diameter of 5 mm before treatment); for the concentric stenosis of the right main bronchus (4 mm) and left main bronchus (2 mm), progressive dilations with rigid instrument up to 12 mm were performed too. After the procedure, the patient started therapy with systemic corticosteroids with benefit on the respiratory symptoms.

Subsequently, prednisone dose was increased up to 50 mg/day and remission induction immunosuppressive therapy with rituximab (375 mg/m^2^/week, 4 intravenous infusions) was started, with significant amelioration of respiratory symptoms. Glucocorticoids gradually tapered down and remission maintenance therapy with rituximab 500 mg was administered at 6 months.

**Figure 2:**
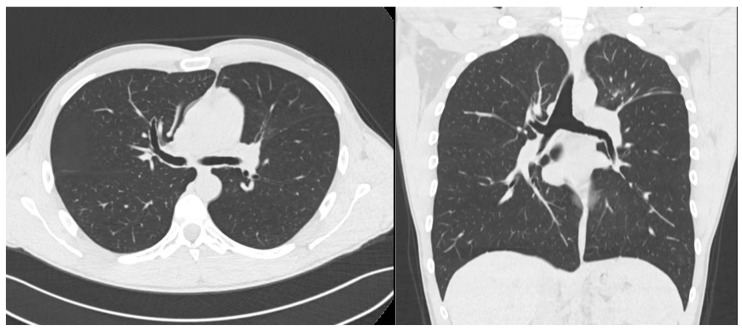
From left to right: Increased left and right main bronchus diameter after endoscopic and systemic treatment (CT scan axial projection). Increased left and right main bronchus diameter after endoscopic and systemic treatment (CT scan coronal projection).

The 3-month follow-up CT scan of neck and thorax (Figure 2) showed increased tracheal airway diameter, generalized improvement in caliber in the right bronchial hemisystem and increased caliber of the left main bronchus but with residual irregular wall thickening still appreciable; the left upper lobar bronchus was still completely obstructed.

After 6 months of treatment, an endoscopic control was performed too, with evidence of marked improvement in tracheobronchial patency bilaterally and no significant signs of inflammation.

After 2 years, the patient is still on remission maintenance treatment with rituximab 500 mg 6-monthly infusions and prednisone 5 mg/day. He started regular ORL, nephrological and pneumological follow-up. No disease relapse, neither systemic nor ENT-limited, occurred during follow-up, with partial resolution of TBS.

## 3. Discussion

Granulomatosis with polyangiitis (GPA), previously known as Wegener granulomatosis, is a rare multisystem autoimmune disease of unknown etiology. GPA is one of the antineutrophil cytoplasmic antibody (ANCA)–associated vasculitis; it can affect the upper and lower respiratory tract, ears, nose, skin, kidneys, and peripheral nervous system. Its main characteristics include necrotizing granulomatous inflammation and pauci-immune vasculitis in small- and medium-sized blood vessels.

A thorough clinical, biochemical, radiographic, and histological evaluation is necessary when there is a high suspicion of GPA disease. To ascertain the locations and degree of illness involvement, clinical examination is essential [1].

In 2022, the ACR/European Alliance of Associations for Rheumatology (EULAR) provided weighted criteria in order to classify and differentiate ANCA-associated vasculitis. This new classification provided a 93% sensitivity and 94% specificity and contains the following parameters:

- Bloody nasal discharge, nasal crusting, or sinonasal congestion (+3);
- Cartilaginous involvement (+2);
- Conductive or sensorineural hearing loss (+1);
- Cytoplasmic ANCA or anti-PR3 ANCA positivity (+5);
- Pulmonary nodules, mass, or cavitation on chest imaging (+2);
- Granuloma or giant cells on biopsy (+2);
- Inflammation or consolidation of the nasal/paranasal sinuses on imaging (+1);
- Pauci-immune glomerulonephritis (+1);
- Perinuclear ANCA or anti-MPO ANCA positivity (−1);
- Eosinophil count more than 1 × 10^9^ cells/L (−4).

A patient diagnosed with small- or medium-vessel vasculitis may be classed as having GPA if the cumulative score is 5 or more points after ruling out other illnesses that could mimic vasculitis [2].

Fever, malaise, weight loss, and myalgia are common nonspecific signs of systemic illness that accompany GPA.

Regarding respiratory involvement, upper respiratory tract symptoms such as sinus and nasal discomfort, purulent nasal discharge, nasal ulcerations, epistaxis, and otitis media are experienced by about 90% of patients. A saddle nose deformity may result from nasal inflammation-induced nasal bridge collapse or septal perforation. The lower respiratory system is frequently affected as well; symptoms include tracheal obstruction, cough, hemoptysis, dyspnea, and occasionally pleuritic chest discomfort. Bilateral or unilateral pulmonary infiltrates are seen in nearly half of patients at first presentation. Additionally, 15% to 20% of cases have been observed to have pleural effusion. One major cause of morbidity and death is diffuse pulmonary hemorrhage [3].

Around 16–23% of patients affected by granulomatosis with polyangiitis (GPA) could develop tracheobronchial stenosis (TBS), which can frequently be fatal and cause significant functional impairment.

Tracheobronchial stenosis can appear on its own, and its course might not be associated with antineutrophil cytoplasmic autoantibody (ANCA) titers or other GPA disease activity.

Management of GPA-associated tracheobronchial stenosis is so complex that a multidisciplinary approach including evaluation by and discussion amongst interventional pulmonologist, otolaryngologists, rheumatologists, head and neck surgery, and thoracic surgery experts is therefore mandatory [4].

Previous research showed that over half of patients needed repeated endoscopic procedures, and systemic therapies were often ineffective. Subglottic stenosis and bronchial stenosis pose a therapeutic challenge because it is still uncertain which endoscopic procedures and optimal systemic medications will be the most effective, as well as the ideal scheduling and association strategy for these procedures [5].

Concerning pharmacological treatment for GPA, it consists of two phases: both induction and maintenance of remission. Immunosuppressive therapy aims to induce long-term remission, which may interfere with the clinical recurrence of disease activity (relapse). Treatment for remission–maintenance is started when illness symptoms have been controlled [6,7].

With respect to endoscopic treatments, no widely standardized therapeutic approaches are accepted yet, such as choice of bronchoscopic technique or when to refer for surgical management. For GPA patients with TBS, a variety of endoscopic treatments have been proposed. Both tracheal and bronchial stenosis have been treated with conservative laser surgery and local dilatation. While stenting was a common endoscopic treatment option for patients with bronchial stenosis, it must be avoided in this inflammatory setting. In contrast, the use of local steroid injections was limited to subglottic stenosis. On the other hand, cryotherapy and topical mitomycin have hardly ever been employed [4,8].

Regarding the treatment of GPA patients with tracheal and bronchial stenosis, there is no clear consensus. Because airway lesions frequently do not respond to pharmacological treatment with corticosteroids or immunosuppressive drugs alone, endoscopic procedures like cryotherapy, balloon dilatation, or stent implantation are required to control the condition. Because of the high likelihood of restenosis, the benefits of endoscopic intervention may be temporary. Comparing patient results according to the preferred treatment mode would provide a more standardized and transferable method [8]

TBS in GPA has not been the subject of many papers. The most common stenosis type documented is subglottic stenosis (SGS); however, the few publications on SGS have primarily been case reports. Even less research has been performed on BSs, and less is known about their clinical characteristics, epidemiology, and therapeutic outcomes.

Here, below we report a brief literature review of GPA-related subglottic stenosis focusing on management and outcomes:

- Girard released a case series in 2016 that included 10 patients with ≥1 bronchial stenosis (BS) and 16 patients with solitary SGS. The median age at GPA diagnosis was 32 years old (3:13 and 28 years, respectively, for SGS patients), and the male/female sex ratio was 9:17. Overall, 62% of patients had at least one stenosis relapse despite receiving standard GPA therapy, compared to 81% of SGS patients, who had one to eight relapses overall. Future relapses were not averted by any of the several endoscopic or systemic therapies. Out of the seven patients treated, four out of six with BS(s) and one with SGS responded well to cyclophosphamide induction therapy. In 3/4 of SGS patients, rituximab produced remission following numerous relapses. The effectiveness of endoscopic procedures (dilation, laser, corticosteroid injection, etc.) was only temporary. Independent of TBSs, other GPA symptoms also relapsed. Acute respiratory distress syndrome claimed the life of one SGS patient. To be more specific, a total of 51 TBS relapses (42 SGS and 9 BS relapses) occurred in 16 patients (62%) who had at least one TBS relapse. While the GPA was in remission, 8 (89%) of the 9 BS and 22/42 (52%) of the SGS relapsed. With the exception of four SGS relapses that coincided with joint or eye involvement (scleritis), ENT disease was the only GPA manifestation throughout the remaining TBS relapses. Of the 16 TBS-relapsing patients, 10 (63%; 1 with BSs and 9 with SGS) maintained systemic GPA remission from the time of TBS diagnosis to the end of follow-up. Of the 26 patients, 6 (23%) experienced relapses of systemic disease, primarily ENT, but no TBS recurrence. As the sole patient with both BSs and SGS, patient 1 had six BS relapses, including five in the left main bronchus, and six SGS relapses, three of which happened without any accompanying BS relapse. Among SGS patients, patient 22 passed away from acute respiratory distress brought on by obstructive SGS following her first relapse, while patient 17 lost her sight following two relapses. Following first treatment or relapse management, the remission rates were 26% for SGS patients, 53% for BS patients, and 32% for all patients. The mean time between their subsequent relapses was 16, 8, and 18 months, respectively. Overall, 46% of cases treated solely medically without concurrent local procedures experienced remission (75% in the case of BS, 35% in the case of SGS). Further relapses were avoided in 24% of instances by combined therapy, 25% by local treatment alone, and 0% by BS patients [8].
- In 2015, a French nationwide retrospective study that included 47 patients with GPA-related TBS was conducted. Patients with TBS were younger, more likely to be female, and less likely to have kidney, ophthalmic, or gastrointestinal involvement as well as mononeuritis multiplex than those without the condition. There were 137 tracheal and 50 bronchial endoscopic procedures, primarily endoscopic dilatation, conservative laser surgery, and local steroid injection, with stenting occurring less frequently. The cumulative rate of endoscopic treatment failure following the initial endoscopic surgery was 49% at 1 year, 70% at 2 years, and 80% at 5 years. A shorter time from GPA diagnosis to endoscopic operation [hazard ratio (HR) 1.08 (95% CI 1.01, 1.14); *p* = 0.01] and bronchial stenosis [HR 1.96 (95% CI 1.28, 3.00); *p* = 0.002] were factors substantially linked to a higher cumulative frequency of treatment failure. A decreased cumulative incidence of treatment failure was linked to a prednisone dose of at least 30 mg per day at the time of the surgery [HR 0.53 (95% CI 0.31, 0.89); *p* = 0.02]. The authors came to the conclusion that TBS has a high rate of restenosis and is a severe and refractory symptom. Better event-free survival is linked to higher time between GPA diagnosis and bronchoscopic intervention, as well as high-dose systemic CSs throughout the surgery. On the other hand, compared to subglottic stenosis, bronchial stenoses are linked to a greater incidence of restenosis [8].
- In 2024, Ashjan Almuhanna published the first systematic review of this topic. The Preferred Reporting Items for Systematic Reviews and Meta-Analyses standards have been followed in this process. The review covered 224 cases in all, with patients ranging in age from 16 to 81. There were only 65 (29.5%) male patients, and 53 patients (24%) had preoperative immunosuppression administered. Dilatation of the subglottic area was the technique most often employed among the ten studies that examined endoscopic therapies for SGS in GPA; nine of these studies used an endoscopic balloon for dilatation, whereas only one study employed dilatation tracheostomy for management. Only one trial used an extra application of mitomycin-C, although all cases required concurrent interlesional steroid injection. Four trials showed that laser surgery and topical drug administration might resect the SGS segment. The most often used lasers were CO_2_, KTP, and nd-YAG lasers.

In every study, endoscopic methods demonstrated instant improvement without the need for a tracheostomy. Pneumothorax in one patient and lesion in the tracheal mucosa in two cases were the only endoscopic surgery problems reported. Studies that used endoscopic procedures included direct injection of corticosteroids into the lesion, dilatation and injection, and laser excision of the stenotic portion. Patients received the intratracheal dilatation–injection method (IDIT), which was the most common intervention in a number of investigations, as reported in the literature. The best results were obtained by patients who received concurrent systemic immunosuppression, but most patients had better outcomes in terms of long-term median survival, subsequent need for follow-up procedures, and requirement for tracheostomies because multiple patients were able to be decannulated after using this technique. Furthermore, patients who underwent IDIT as their first intervention without any prior surgical procedures (apart from tracheostomy) had better outcomes, with an average of 2.4 procedures, as compared to patients who underwent laser resection or rigid bronchoscopic dilations, which had an average of 4.1 procedures. Traditionally, mitomycin-C is administered over the stenotic region and methylprednisolone is injected locally; both techniques have demonstrated positive results. Reviewing a different method for endoscopic intervention, a novel procedure was carried out in which the stenotic portion was removed submucosally, and the raised mucosal flap was sealed back with the bare areas soaked in mitomycin-C. According to the designated quality of life questionnaire, which contains standardized questions that address postintervention symptom improvement, there was no postprocedural mortality reported, and the quality of life significantly improved at about 85%. When combined with the application of mitomycin-C, the use of a CO_2_ laser in GPA produced highly favorable results. However, prior research indicated that the use of a laser caused stenosis to recur more quickly and widely. This may be related to the laser when there is active inflammation [9].

Finally, an extensive retrospective single-center study published in 2014 involving 44 patients with GPA and airway stenosis reported the assessment and treatment offered by a multidisciplinary team at a university medical center between 1997 and 2012. For every patient, the median observation period from diagnosis was 146 months. Women made up 73% of the patients (n = 34), with a median age of 37.6 years at diagnosis. Following the initial intervention, the median follow-up period was 62.5 months. Thirty patients had lower airway stenosis, while thirty patients had subglottic stenosis. Thirty-nine patients underwent 213 procedures, including laser therapy, balloon and bougie dilatation, and more. In 71 interventions, adjuvant local therapy was employed. For 34 out of 36 cases (97%) airway stability was maintained for 12 months (5 had no surgeries, and 3 had follow-up less than 12 months). Patients had at least 27 months of airway stability following the last intervention documented, with a median gap between treatments of 4.9 months. There were fourteen adverse events (6.6%). Although several operations were needed for the 39 patients who needed intervention, 97% of them were able to maintain airway patency for an extended length of time. It was determined that the adjuvant therapies and procedures were safe. In total, 213 interventions were documented over the course of the follow-up period. Overall, balloon dilatation without adjuvant local therapy was the most prevalent method; however, patients with SGS were treated solely with mitomycin C and a carbon dioxide laser. Four of them had just SGS (median follow-up, 69.1 months; IQR, 33.0–87.7 months), and five (11.4%) did not need intervention. Half of the lesions in the secondary bronchi (23 of 46), two-thirds of the lesions affecting the trachea or major bronchi (18 of 28), and three-quarters of the subglottis lesions (26 of 36) were treated locally. Only three patients underwent a single procedure, and the median number of interventions per patient was two. For significant stenoses, 43 interventions (20.2%) were carried out during active GPA, with a median gap of 4.4 months before the subsequent intervention and 5.2 months between those carried out during GPA remission. The median follow-up length after the final planned intervention was 27 months. Seven of the thirteen patients (30%) who needed tracheostomies during the follow-up period had them decannulated. One patient received a temporary tracheostomy following the 2007 treatment approach modification, and they were decannulated in 22 days. At least one phase of airway stability (≥12 months between treatments) was attained by all but one patient. Overall, patients had a stable patent airway for 45.7% of the follow-up period, with a median of 27.6 without treatments. Three patients with brief follow-up and five patients without interventions were not included in this computation. After more than two years of disease control, most patients who needed additional therapy showed up with airway problems but no obvious reactivation of GPA (11 patients, 12 of 13 treatments). Furthermore, the lesion that needed additional care changed over time in six of these eleven patients. The course of patients in the bronchi group was more refractory. This was also observed in patients with SGS and stenosis in the secondary bronchi only (n = 8; median time with airway stability, 95.7 months) and in the widespread group who had a stenotic lesion in every anatomical area of the bronchial tree (n = 9; median time with airway stability, 55.7 months). According to the published data, the percentage of patients who needed only one surgery was between 11% and 35%. This could be explained by the condition’s refractory character and the lengthy follow-up period in this study. The refractory character of the illness is demonstrated by the number of procedures conducted (n = 213) and the fact that patients had a stable patent airway for slightly under half (45.7%) of their follow-up period. The most refractory course was seen in patients with lower airway stenoses [10].

## 4. Future Perspectives

As our understanding of tracheobronchial stenosis (TBS) in the context of granulomatosis with polyangiitis (GPA) continues to evolve, there are several promising directions for future research and clinical practice that could significantly impact patient management and outcomes. Despite the current challenges in treating GPA-related TBS, advancements in both diagnostic techniques and therapeutic interventions hold the potential to improve the prognosis for these patients:

Early and accurate diagnosis of TBS in patients with GPA is crucial for initiating timely intervention and preventing irreversible damage to the airway. Earlier detection of tracheobronchial involvement, potentially before the onset of significant clinical symptoms, for instance with techniques like positron emission tomography (PET), could provide more detailed visualization of airway changes and inflammatory activity. Additionally, the role of biomarkers, such as specific autoantibodies or cytokine profiles may emerge as key diagnostic tools, helping clinicians to not only identify TBS more effectively but also predict its course and response to treatment [11,12,13].

- While the combination of systemic immunosuppression and endoscopic procedures currently represents the cornerstone of treatment for GPA-related TBS, new and more targeted therapies are being actively explored. Agents that target specific immune pathways are promising in treating GPA, and their potential application to TBS warrants further investigation. These may offer more precise control over the inflammatory process underlying GPA, potentially reducing the frequency and severity of tracheobronchial stenosis. Additionally, the development of novel endoscopic techniques, including the use of laser therapy, balloon dilation, and self-expanding metal stents, continues to advance. The refinement of these techniques could lead to better long-term outcomes for patients. Emerging research on tissue engineering and regenerative medicine also suggests that cellular therapies, such as stem cell-based approaches, might one day play a role in repairing damaged tracheobronchial tissues, potentially providing more durable solutions for stenosis that does not respond to conventional treatments [14,15,16].
- Given the heterogeneity of GPA and its varying effects on the respiratory tract, personalized medicine is likely to become an integral part of managing TBS in these patients. The use of genetic profiling and a deeper understanding of the immunopathogenesis of GPA could allow for more precise identification of patients at risk of developing tracheobronchial stenosis. This would enable clinicians to tailor treatment regimens based on individual genetic and molecular characteristics, improving the likelihood of therapeutic success and minimizing side effects. Moreover, advancements in machine learning may contribute to this process by aiding in the prediction of disease progression and treatment outcomes. This shift towards precision medicine could revolutionize the approach to both diagnosis and treatment, optimizing clinical decision-making [17,18].
- In patients with GPA-related TBS, recurrence of stenosis remains a significant concern, highlighting the need for long-term surveillance and ongoing management. Future research should focus on developing effective strategies for long-term monitoring of the airway, and identifying predictive markers for relapse could allow for pre-emptive interventions, thus preventing exacerbations of stenosis [12,13].
- The complexity of GPA-related TBS underscores the importance of a multidisciplinary approach in both research and clinical care. Collaborative efforts between pulmonologists, rheumatologists, thoracic surgeons, radiologists, and immunologists will be crucial for advancing the management of this condition. Additionally, establishing international registries for patients with GPA-related TBS could facilitate multicenter studies and provide more robust data on treatment efficacy across diverse populations [19].

In summary, while the current management of GPA-related TBS is fraught with challenges, promising developments in diagnostics, targeted therapies, personalized medicine, and multidisciplinary care offer hope for improved outcomes in the future. Ongoing research into these areas will likely refine current approaches and uncover novel treatments, ultimately leading to better long-term quality of life and survival rates for patients suffering from this debilitating condition.

## 5. Conclusions

Tracheobronchial stenosis (TBS) can represent a severe manifestation of granulomatosis with polyangiitis (GPA), leading to significant functional impairment and potentially life-threatening complications. The management of TBS in the context of GPA remains an area of ongoing debate, with no universally agreed-upon treatment protocol. While both systemic immunosuppressive therapy and endoscopic interventions have demonstrated efficacy in alleviating symptoms and managing the condition, the optimal therapeutic approach is still controversial.

The current literature suggests that a combined strategy, integrating both pharmacological treatments aimed at controlling the underlying autoimmune process and endoscopic interventions such as dilation or stent placement, may offer the most promising outcomes for patients. However, the specific balance between these modalities and their sequential or concurrent use remains to be better defined. Furthermore, the evolving nature of GPA and the potential for relapse or progressive airway damage necessitate a tailored, patient-specific approach.

Given the complexity of this condition and the potential for rapid deterioration in affected patients, a multidisciplinary approach is indispensable in both the diagnostic and therapeutic phases. Collaborative decision-making among pulmonologists, rheumatologists, thoracic surgeons, and radiologists is critical to ensuring that both the inflammatory disease process and the mechanical airway obstruction are adequately addressed.

Despite current advances, there remains a gap in understanding the long-term outcomes of various treatment strategies for GPA-related TBS. Larger, multicenter studies are needed to evaluate the efficacy and safety of different management approaches, as well as to identify biomarkers that may guide treatment decisions. Ultimately, further research will be essential to improve the prognosis for patients with GPA-associated tracheobronchial stenosis.

## Data Availability

The data that support the findings of this study are available on request from the corresponding author.
